# Year of study as predictor of loneliness among students of University of Gondar

**DOI:** 10.1186/s13104-019-4274-4

**Published:** 2019-04-29

**Authors:** Baye Dagnew, Henok Dagne

**Affiliations:** 1 Department of Human Physiology, School of Medicine, University of Gondar, P.O. Box 196, Gondar, Ethiopia; 2 Department of Environmental and Occupational Health and Safety, Institute of Public Health, University of Gondar, P.O. Box 196, Gondar, Ethiopia

**Keywords:** Loneliness, Student, Ethiopia

## Abstract

**Objectives:**

Loneliness is individual's subjective sense of lacking familial or social contact to the degree that they wanted. It is responsible for reduced quality of life. The aim of this study was to determine loneliness and its association with year of study among University of Gondar students, 2018/19. Cross-sectional study design was used on 404 Medical and Health Sciences students selected by systematic random sampling. UCLA-R loneliness score was used. A person with a mean value of 42 and above was considered as lonely. After data were collected by self-administered questionnaire, Epi-Data was used for data entry and exported to SPSS version 20.1 for analysis. Variables with p-value of 0.05 and lower were treated as significant factors in multivariable logistic regression.

**Results:**

Prevalence of loneliness was 49.5% (95% CI 44.6–54.4%). Year of study was significantly associated with loneliness [AOR = 2.47, 95% CI (1.65–3.70)]. First-year students were having 2.47 odds of loneliness as compared to second year and above students. Loneliness prevalence was higher in the current study. This must get the attention of higher education institutions, the government and all concerned stakeholders in the education sector to design strategy on preventing and treating loneliness.

## Introduction

Ability of a person for close connection with other people is one of the most important settings of a healthy personality. Loneliness is individual’s personal, subjective sense of lacking social or familial contact to the extent that they wanted [1]. Loneliness affects many physiological processes mainly due to hypothalamic–pituitary–adrenal axis disturbances leading to poor daily performances by inducing sleep in individuals [2]. It affects a person in his all lifespan more likely to occur under circumstances like prolonged absence from home or loss of a significant-other [3]. In Ankara University students 60.2% of students experienced loneliness which was associated with the need for economic support, social interaction and romantic relationship [4]. A study from Maragheh University students indicated prevalence of moderate and severe loneliness as 50.5% and 31.6%, respectively where sex was predictor [5]. Loneliness varies in different settings. About 10.5% loneliness prevalence was seen in general population of Western Mid-Germany which was higher in females and without partner [6]. Loneliness is predicted by lower family wealth, living in a low or lower middle-income country, low organized religious activity. Lonely students self-reported poor subjective health status, sleeping problems, short sleep duration and tobacco use [7]. Male students had higher levels of Loneliness than females as seen in Anadolu University [8]. The transition from high school to University often causes much stress for most students. In the new University environment, students often face various interpersonal, social, and academic demands, each of which could potentially create stressful situations for most of them which can lead to specific problems in adjustment [9]. Being bound in a romantic relationship is protective in a study done in China, Thai international students and Turkey [10, 11]. Number of semesters was significantly associated with loneliness with lower loneliness in those staying for longer semesters in university [5, 8]. Social isolation is found to be correlated with loneliness that ended up with depression among adults as indicated by a study done in London [12]. Quality of life is affected negatively in persons with loneliness [13] pushing adolescents to use life-threatening substances such as Marijuana [14]. Loneliness is responsible for most depressive symptoms [8] and may lead to cognitive impairment [15]. A study in China revealed, loneliness is not only the cause for the poor quality of life but also increases mortality [16]. Loneliness is significantly associated with divorced status, social support and psychological wellbeing [17]. Individuals with loneliness had reduced out of home physical activities [18]. Mental disturbances and other unhealthy states are common in lonely individuals [19]. Freshman students had problems to adjust themselves into new environment as evidenced in University students at Dila, Ethiopia [20]. Cultural backgrounds affect loneliness which may increase loneliness in some and decreases in others [21]. Increased recognition of loneliness as a risk factor for adverse psychological and physical health outcomes has elevated interest in interventions to reduce chronic loneliness [22]. As many scholars agreed, loneliness is one of the major predisposing factors for disturbed quality of life and poor productivity. As far as our knowledge is concerned such kind of study was not conducted in Ethiopia. Because of this, the investigators had been interested in conducting this research. After completion, this might help to add additional information besides the existing literature to the scientific community. The study population will be benefited from the study findings since the results will help the institutions to design interventional strategies for loneliness and related issues. The major objective of this study was assessing loneliness and determining if loneliness is predicted by year of study among University students, University of Gondar, 2018/19.

## Main text

### Methods

#### Study area and period

The study was conducted at University of Gondar, Ethiopia. The University of Gondar has been dedicated to educating students for more than 64 years and serving the community by delivering clinical services in its comprehensive specialized teaching hospital. The study was conducted from Oct 01 to Nov 30, 2018.

Study design: Institution-based cross-sectional study design was employed.

#### Source population

All regular students of University of Gondar engaged in learning process in 2018/19. A total of 2358 regular undergraduate students were found in the College of Medicine and Health Sciences.

#### Study population

Regular undergraduate Medical and Health Science students of University of Gondar who were engaged in the learning process in the academic calendar of 2018/19 those present at the time of data collection period.

#### Eligibility criteria

Inclusion criteria: All regular undergraduate 1st year to graduating class students of University of Gondar College of Medicine and Health Science were included.

Exclusion criteria: students with a severe illness that limits them to fill questions were excluded.

#### Sample size determination

The sample size (n) was calculated by using a single population proportion formula by using assumptions; magnitude of loneliness (p) among students in University of Gondar = 50% (since there was no previous study in Ethiopia, we preferred to use maximum sample size), 95% level of confidence and 5% margin of error (d).

$$\begin {aligned}{\varvec{n}}&=\frac{{\left({{\varvec{z}}}_{\frac{{\varvec{a}}}{2}}\right)}^{2}\times {\varvec{p}}\times (1-{\varvec{p}})}{{{\varvec{d}}}^{2}} \\ &=\frac{{\left(1.96\right)}^{2}\times 0.5\times (1-0.5)}{{(0.05)}^{2}}=384 \end {aligned}$$

By adding 5% (expected non-response rate), the minimum calculated sample was 404.

#### Variables of the study

Dependent variable**:** Loneliness (Yes/No).

Independent variables: Age, sex, residence before coming to University, marital status, lifestyle (Khat chewing, cigarette smoking), health status, romantic love engagement, year of study and monthly pocket income, current disease status.

#### Operational definitions

Loneliness: A person with a mean score of 42 and above out of 80 total standard loneliness score was regarded as having loneliness.

Current disease status: If a student is faced with any sort of disease in the past 1 month from data collection period, he/she is referred to having current disease.

Year of study: The education level of students in the University of Gondar during data collection.

#### Data collection instrument and procedure

The structured pretested self-administered questionnaire was used. Revised University of California Los Angeles Loneliness scale (UCLA-R) was used to collect data about subjective feelings of loneliness [23]. A UCLA-R scale has 20-item questions with four alternatives (Never = 1, rarely = 2, Sometimes = 3, and Often = 4) ranging from 20 (lowest score) to 80 (highest score). First, the adapted questionnaire was prepared in English and translated to Amharic and then, translated back to English by another person to check its consistency and wording. The cut-off point for describing loneliness was calculated by the mean and the score above the mean value was indicative of loneliness. Two supervisors participated in the data collection.

#### Data quality management/control

One day training about the ethical issues, the purpose of the study and data collection techniques were given for supervisors. Pretest was performed on 40 students outside of the study area. During data collection, close supervision, spot-checking and reviewing the completed questionnaire were done by the supervisors and principal investigator on daily basis. Data clean up and cross-checking was done before analysis. Cronbach’s alpha was done with the result of 0.78 which is acceptable [24].

#### Data processing and analysis

Data were edited, coded and entered into epidemiological data (EPI-DATA) version 3.1 and exported to Statistical package for Social Sciences (SPSS) version 20 for analysis. Descriptive statistics were presented in frequency tables with percentage. Students who scored above a mean value (42 and above) were considered as facing loneliness. The bivariable analysis was done for loneliness and independent variables to check for crude association. Variables with a p-value of < 0.2 in bivariable analysis were candidates and entered to multivariate logistic regression analysis to identify the independent determinants of loneliness. Both Crude Odds Ratio (COR) and the Adjusted Odds Ratio (AOR) with a corresponding 95% confidence interval (CI) were computed to show the strength of association. Hosmer and Lemeshow goodness of fit test was checked (p-value > 0.05). Variables with p-value of < 0.05 in the multivariate logistic regression analysis were taken as statistically significant.

### Result

#### Description of study participants

A total of 404 students participated with 100% response rate. Of these, 242 (59.9%) were males and 238 (58.9%) were below 21 years. 127 (31.4%) students were actively engaged in romantic relationship. Only 3.2% and 3% of students were currently chewing chat and smoke cigarette respectively (Table 1).

**Table 1:** Socio-demographic property of participants, University of Gondar, 2018 (n = 404)

Study variables	Categories	Frequency (N)	Percentage (%)
Sex	Male	242	59.9
	Female	162	40.1
Age	20 years and lower	238	58.9
	21 years and above	166	41.1
Religion	Orthodox	318	78.7
	Muslim	31	7.7
	Protestant	45	11.1
	Catholic	8	2.0
	Others	2	0.5
Engaged in romantic love	Yes	127	31.4
	No	277	68.6
Residence before coming to university	Urban	246	60.9
	Rural	158	39.1
Current Khat chewing	Yes	13	3.2
	No	391	96.8
Current cigarette smoking	Yes	12	3.0
	No	392	97.0
Current disease status	Yes	199	49.3
	No	205	50.7
Management of subjective loneliness	Go to religious places	251	62.1
	Go to recreational places	60	14.9
	Go to sleep	70	17.3
	Watching TV/Film	23	5.7
Year of study	First year	226	55.9
	Second year & above	178	44.1
Monthly pocket money	50–400	111	27.5
	401–500	106	26.2
	501–1000	134	33.2
	Above 1000	53	13.1
Marital status	Single	392	97.0
	Married	12	3.0

#### The magnitude of loneliness among study participants

From 404, participants 200 (49.5%) students had a score above mean fore loneliness indicating these had loneliness. Within group, females were lonelier than males but intergroup comparison indicated more males had loneliness. Those who had not engaged in a romantic relationship were more lonely (52%) as compared to those who were not engaged. The magnitude of loneliness was higher in those rural residents before coming to the university, current khat chewers and cigarette smokers and students with the age group of 21 years and above (Table 2).

**Table 2:** Associated factors of loneliness in multivariable logistic regression among study participants, University of Gondar, 2018 (n = 404)

Variable	Categories	Loneliness status				COR	AOR (95% CI)
		Lonely		Not lonely		OR (95% CI)	OR (95% CI)
		N	(%)	N	(%)		
Sex	Male	113	46.7	129	53.3	1	
	Female	87	53.7	75	46.3	1.324 (0.89–1.97)	1.3 (0.87–1.98)
Actively engaged in romantic friendship	Yes	56	44.1	71	55.9	1	
	No	144	52.0	133	48.0	1.37 (0.90–2.09)	1.3 (0.85–2.02)
Residence before coming to university	Urban	117	47.6	129	52.4	1	
	Rural	83	52.5	75	47.5	1.22 (0.82–1.82)	
Current khat chewing	Yes	7	53.8	6	46.2	1.19 (0.39–3.63)	
	No	193	49.4	198	50.6	1	
Current cigarette smoking	Yes	6	50	6	50.0	1.02 (0.32–3.22)	
	No	194	49.5	198	50.5	1	
Current disease status	Yes	102	51.3	97	48.7	1.15 (0.77–1.69)	
	No	98	47.8	107	52.2	1	
Age in years	20 & lower	120	50.4	118	49.6	1.09 (0.74–1.63)	
	21 & above	80	48.2	86	51.8	1	
Year of study	First	134	59.3	92	40.7	2.47 (1.67–3.7)	2.47 (1.65–3.70)a
	2nd & above	66	37.1	112	62.9	1	1
Monthly pocket money in ETB	50–400	53	47.7	58	52.3	0.82 (0.42–1.5)	
	401–500	46	43.4	60	56.6	0.68 (0.35–1.33)	
	501–1000	73	54.5	61	45.5	1.07 (0.56–2.02)	
	> 1000	28	52.8	25	47.2	1	
Marital status	Single	192	49.0	200	51.0	0.48 (0.14–1.62)	
	Married	8	66.7	4	33.3	1	

CI: confidence interval; COR: Crude odds ratio; AOR: Adjusted odds ratio; ^a^Indicates significantly associated variable with loneliness in multivariable analysis

#### Factors associated with Loneliness

All independent variables were tested for crude association with loneliness by binary logistic regression. Of the tested variables; Sex, Active engagement in a romantic relationship and year of study had a p-value of < 0.2 and hence entered to multivariable logistic regression with a backward Likelihood ratio (LR) to find out significantly associated factors of loneliness. In multivariable logistic regression, only year of study was significantly associated (p-value = 0.00). After controlling other variables constant, first-year students were 2.47 times more likely to develop loneliness than second year and above [AOR = 2.47, 95% CI (1.651–3.701)] (Table 2).

### Discussion

Findings of the current study revealed the magnitude of loneliness among University of Gondar students being 49.5% [95% CI 44.6–54.4%] which is a major public health problem with females more affected than males in proportion. This is lower than a report in Turkey University students which disclosed 60.2% loneliness [4]. In contrary, the result of this study showed a higher prevalence of loneliness as compared to a study in Mid-Germany that reported a 10.5% [6] and 10.2% (meta-analysis study) prevalence of loneliness [25]. This difference could be associated with the nature of study participants, sample size and sociocultural differences. No more articles were found about the magnitude of loneliness that limited us to compare our study findings.

As indicated in different studies, there were different factors contributing to loneliness like family residence, romantic relationship (partnership), sex and family wealth (income) [2]. In this study amongst the covariates tested in binary logistic regression, only sex, Active engagement in a romantic relationship and year of study had p-value less than 0.2. Of these variables year of study was significantly associated with loneliness; being first year is highly risky for having loneliness than second year and above students. The association of loneliness among first year (freshman) students could be due to the short duration of stay in the University to adapt the new environment which is supported by a study done in Washington University that indicated leaving home for college made students susceptible to experience loneliness [26]. Being in a romantic relationship is preventive to loneliness as reported in Germany [27] even though it is not significantly associated in our study. Our study findings indicated a major proportion of students were affected by loneliness which gives insight for University authorities to work on loneliness preventive and therapeutic strategies so as to reduce difficulties of students in the learning environment. This study revealed a higher magnitude of loneliness which is mainly found in first-year students. Being first year is more prone to loneliness. This gives a clue for officials to include coping strategies for loneliness in University students. The investigators would like to recommend researchers to conduct more qualitative studies to find out concrete information on loneliness. Besides this, Universities has to plan on how to put students in a comfortable situation while they are separating from their family. Above all guidance and counseling institutions in Universities has to approach students to prevent and treat loneliness.

## Limitation of the study

This study was undertaken using a standardized tool which helped to compare with previous studies. The generalizability and validity of the study result is good. However, this study was not without limitations. The potential limitations include the nature of the study design which cannot be used to show cause-effect relationship. In addition the self-reported nature of the study tool might result in social desirability bias.

## Abbreviations

AOR: adjusted odds ratio
CI: confidence interval
COR: crude odds ratio
Epi-Data: epidemiological data
SPSS: Statistical package for social sciences
UCLA-R: Revised University of California Loneliness Assessment scale
